# Supplementary material for: Does the Phytochemical Diversity of Wild Plants Like the Erythrophleum genus Correlate with Geographical Origin?
Source: Molecules. 2021 Mar 17;26(6):1668. doi: 10.3390/molecules26061668 (PMC8002556; doi:10.3390/molecules26061668)
Supplement: Supplementary file 1 [file molecules-26-01668-s001.pdf]

Supplementary material

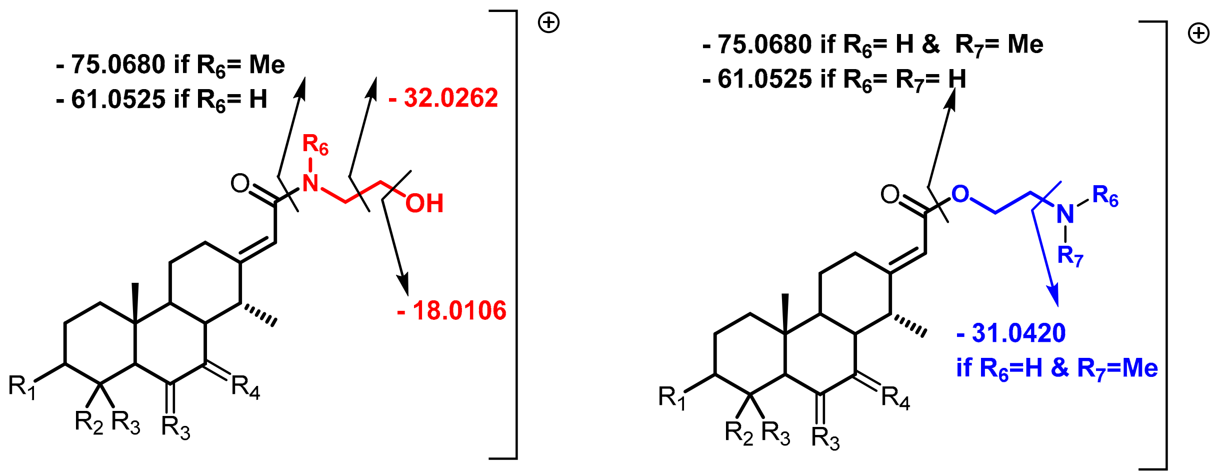

Figure S1: Fragmentation pattern characteristics of amide compounds (**A**) or ester compound (**B**). The amide compound is characterized by a neutral loss of H_2O and MeOH, while the ester compound is characterized by a neutral loss of solely $NH(Me)_2$
